# Supplementary material for: Herbivore and pollinator body size effects on strawberry fruit quality
Source: PLoS One. 2024 Jun 25;19(6):e0305370. doi: 10.1371/journal.pone.0305370 (PMC11198852; doi:10.1371/journal.pone.0305370)
Supplement: S1 Table — Fixed effects: Lygus size ~ Urban cover at 750m + Sex. (DOCX) [file pone.0305370.s001.docx]

**S1 Table : Results of linear mixed-effects model fit by REML comparing *Lygus* pronotal width and urban cover at 750 meters.**

|  | Value | Std. Error | DF | t-value | p-value |
| --- | --- | --- | --- | --- | --- |
| (Intercept) | 1.9882540 | 0.0277504 | 56 | 71.64771 | **0.0000** |
| Urban at 750 | 1.2183231 | 0.4930992 | 8 | 2.47075 | **0.0387** |
| Sex (M) | -0.0716182 | 0.0311261 | 56 | -2.30090 | **0.0251** |

Fixed effects: *Lygus* size ~ Urban cover at 750m + Sex

**​​**
