# Supplementary material for: Herbivore and pollinator body size effects on strawberry fruit quality
Source: PLoS One. 2024 Jun 25;19(6):e0305370. doi: 10.1371/journal.pone.0305370 (PMC11198852; doi:10.1371/journal.pone.0305370)
Supplement: S2 Table — Fixed effects: Lygus size ~ Natural cover at 750m + Sex. (DOCX) [file pone.0305370.s002.docx]

**S2 Table: Results of linear mixed-effects model fit by REML comparing *Lygus* pronotal width and natural forest cover at 750 meters.**

|  | Value | Std. Error | DF | t-value | p-value |
| --- | --- | --- | --- | --- | --- |
| (Intercept) | 2.1162251 | 0.04468308 | 56 | 47.36078 | **0.0000** |
| Nat cover at 750 | -0.3404832 | 0.15765017 | 8 | -2.15974 | 0.0628 . |
| Sex (M) | -0.0817943 | 0.03145900 | 56 | -2.60003 | **0.0119** |

Fixed effects: *Lygus* size ~ Natural cover at 750m + Sex
