# Supplementary material for: Herbivore and pollinator body size effects on strawberry fruit quality
Source: PLoS One. 2024 Jun 25;19(6):e0305370. doi: 10.1371/journal.pone.0305370 (PMC11198852; doi:10.1371/journal.pone.0305370)
Supplement: S3 Table — Fixed effects: Strawberry fruit weight (g) ~ pollination treatment + Lygus presence/absence. (DOCX) [file pone.0305370.s003.docx]

**S3 Table: Results of linear mixed-effects model fit by REML comparing strawberry fruit weight with pollination treatment and tarnished plant bug presence/absence.**

|  | Value | Std. Error | DF | t-value | p-value |
| --- | --- | --- | --- | --- | --- |
| (Intercept) | 6.718101 | 0.5145522 | 198 | 13.056209 | **0.0000** |
| Pollination treatment | 3.675543 | 0.5960672 | 198 | 6.166322 | **0.0000** |
| TPB presence (absent) | -0.095231 | 0.5960672 | 198 | -0.159765 | 0.8732 |

Fixed effects: Strawberry fruit weight (g) ~ pollination treatment + *Lygus* presence/absence
