# Supplementary material for: Herbivore and pollinator body size effects on strawberry fruit quality
Source: PLoS One. 2024 Jun 25;19(6):e0305370. doi: 10.1371/journal.pone.0305370 (PMC11198852; doi:10.1371/journal.pone.0305370)
Supplement: S1 Fig — Fruit weight by size of tarnished plant bug the plant was exposed to for 24 hours. Red points are those flowers visited by large bees (first panel), green points are those visited by small bees (second panel), and blue are unvisited flowers (third panel). Trendlines = linear regressions, shaded area = 95% confidence area. (DOCX) [file pone.0305370.s004.docx]

**S4 Figure: Tarnished plant bug size by fruit weight and pollination**

**
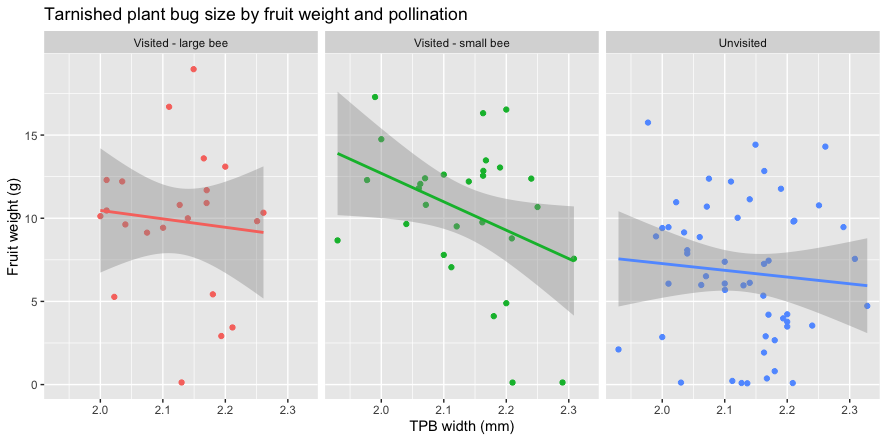
**

Legend: Fruit weight by size of tarnished plant bug the plant was exposed to for 24 hours. Red points are those flowers visited by large bees (first panel), green points are those visited by small bees (second panel), and blue are unvisited flowers (third panel). Trendlines = linear regressions, shaded area = 95% confidence area.
