# Supplementary material for: Herbivore and pollinator body size effects on strawberry fruit quality
Source: PLoS One. 2024 Jun 25;19(6):e0305370. doi: 10.1371/journal.pone.0305370 (PMC11198852; doi:10.1371/journal.pone.0305370)
Supplement: Attachment — Submitted filename: PONE-D-23-40097 A. Monfared Final.pdf [file pone.0305370.s005.pdf]

PLOS ONE

The impact of land use change on intraspecific variation in pollinators and herbivores and its effect on strawberry production

--Manuscript Draft--

**Manuscript Number:**	PONE-D-23-40097
**Article Type:**	Research Article
**Full Title:**	The impact of land use change on intraspecific variation in pollinators and herbivores and its effect on strawberry production
**Short Title:**	The impact of land use change on intraspecific variation in pollinators and herbivores in strawberry crops
**Corresponding Author:**	Annika Leigh Salzberg, B.S.<br>Cornell University College of Agriculture and Life Sciences<br>Ithaca, NY UNITED STATES
**Keywords:**	functional traits; landscape composition; pollination; herbivory; ecosystem services; Fragaria x ananassa
**Abstract:**	Land use change affects both pollinator and herbivore populations with consequences for crop production. Recent evidence also shows that land use change affects insect traits, with intraspecific body size of pollinators changing across landscape gradients. However, the consequences on crop production of trait changes in different plant interactors have not been well-studied. We hypothesized that changes in body size of key species can be enough to affect crop productivity, and therefore looked at how the field-realistic variation in body size of both an important pollinator,Bombus impatiens, and a key pest herbivore,Lygus lineolaris, can affect fruit size and damage in strawberry. First, we determined if herbivores, like pollinators, also vary in body size along land use gradients; and second, we tested under controlled conditions how the individual and combined changes in size of an important pollinator and a key herbivore pest affect strawberry fruit production. The key herbivore pest was smaller in landscapes with more natural and semi-natural habitat, confirming that herbivore functional traits can vary along a land use gradient. Additionally, herbivore size, and not pollinator size, marginally affected fruit production - with plants exposed to larger pests producing smaller fruits. Our findings suggest that land use changes at the landscape level affect crop production not just through changes in the taxonomic diversity of insect communities that interact with the plant, but also through changes in body size traits.
**Order of Authors:**	Annika Leigh Salzberg, B.S.
	Heather Grab, PhD
	Casey Hale, B.S.
	Katja Poveda, PhD
**Additional Information:**	
**Question**	**Response**
**Financial Disclosure**<br><br>Enter a financial disclosure statement that describes the sources of funding for the work included in this submission. Review the submission guidelines for detailed requirements. View published research articles from *PLOS ONE* for specific examples.	Yes

Powered by Editorial Manager® and ProduXion Manager® from Aries Systems Corporation

This statement is required for submission and **will appear in the published article** if the submission is accepted. Please make sure it is accurate.<br><br>**Funded studies**<br>Enter a statement with the following details:<br>• Initials of the authors who received each award<br>• Grant numbers awarded to each author<br>• The full name of each funder<br>• URL of each funder website<br>• Did the sponsors or funders play any role in the study design, data collection and analysis, decision to publish, or preparation of the manuscript?<br><br>Did you receive funding for this work?	
Please add funding details.<br>as follow-up to "**Financial Disclosure**<br><br>Enter a financial disclosure statement that describes the sources of funding for the work included in this submission. Review the submission guidelines for detailed requirements. View published research articles from *PLOS ONE* for specific examples.<br><br>This statement is required for submission and **will appear in the published article** if the submission is accepted. Please make sure it is accurate.<br><br>**Funded studies**<br>Enter a statement with the following details:<br>• Initials of the authors who received each award<br>• Grant numbers awarded to each author<br>• The full name of each funder<br>• URL of each funder website<br>• Did the sponsors or funders play any role in the study design, data collection and analysis, decision to publish, or preparation of the manuscript?<br><br>Did you receive funding for this work?"	This work was supported by USDA-NIFA (https://www.nifa.usda.gov/) grant #2019-67013-29367 received by Katja Poveda. The funder did not play a role in study design, data collection or analysis, nor decision to publish/preparation of the manuscript.

Powered by Editorial Manager® and ProduXion Manager® from Aries Systems Corporation

Please select the country of your main research funder (please select carefully as in some cases this is used in fee calculation).<br>as follow-up to "**Financial Disclosure**<br><br>Enter a financial disclosure statement that describes the sources of funding for the work included in this submission. Review the submission guidelines for detailed requirements. View published research articles from *PLOS ONE* for specific examples.<br><br>This statement is required for submission and **will appear in the published article** if the submission is accepted. Please make sure it is accurate.<br><br>**Funded studies**<br>Enter a statement with the following details:<br>• Initials of the authors who received each award<br>• Grant numbers awarded to each author<br>• The full name of each funder<br>• URL of each funder website<br>• Did the sponsors or funders play any role in the study design, data collection and analysis, decision to publish, or preparation of the manuscript?<br><br>Did you receive funding for this work?"	UNITED STATES - US
**Competing Interests**<br><br>Use the instructions below to enter a competing interest statement for this submission. On behalf of all authors, disclose any competing interests that could be perceived to bias this work—acknowledging all financial support and any other relevant financial or non-financial competing interests.<br><br>This statement is required for submission and **will appear in the published article** if the submission is accepted. Please make sure it is accurate and that any funding	The authors have declared that no competing interests exist.

sources listed in your Funding Information later in the submission form are also declared in your Financial Disclosure statement.<br><br>View published research articles from *PLOS ONE* for specific examples.<br><br>**NO authors have competing interests**<br><br>Enter: *The authors have declared that no competing interests exist.*<br><br>**Authors with competing interests**<br><br>Enter competing interest details beginning with this statement:<br><br>*I have read the journal's policy and the authors of this manuscript have the following competing interests: [insert competing interests here]*<br><br>* typeset	
**Ethics Statement**<br><br>Enter an ethics statement for this submission. This statement is required if the study involved:<br><br>• Human participants<br>• Human specimens or tissue<br>• Vertebrate animals or cephalopods<br>• Vertebrate embryos or tissues<br>• Field research<br><br>Write "N/A" if the submission does not require an ethics statement.<br><br>General guidance is provided below. Consult the submission guidelines for detailed instructions. **Make sure that all information entered here is included in the Methods section of the manuscript.**	N/A

**Format for specific study types**<br><br>**Human Subject Research (involving human participants and/or tissue)**<br>• Give the name of the institutional review board or ethics committee that approved the study<br>• Include the approval number and/or a statement indicating approval of this research<br>• Indicate the form of consent obtained (written/oral) or the reason that consent was not obtained (e.g. the data were analyzed anonymously)<br><br>**Animal Research (involving vertebrate animals, embryos or tissues)**<br>• Provide the name of the Institutional Animal Care and Use Committee (IACUC) or other relevant ethics board that reviewed the study protocol, and indicate whether they approved this research or granted a formal waiver of ethical approval<br>• Include an approval number if one was obtained<br>• If the study involved *non-human primates*, add *additional details* about animal welfare and steps taken to ameliorate suffering<br>• If anesthesia, euthanasia, or any kind of animal sacrifice is part of the study, include briefly which substances and/or methods were applied<br><br>**Field Research**<br><br>Include the following details if this study involves the collection of plant, animal, or other materials from a natural setting:<br>• Field permit number<br>• Name of the institution or relevant body that granted permission	
**Data Availability**<br><br>Authors are required to make all data underlying the findings described fully available, without restriction, and from the time of publication. PLOS allows rare exceptions to address legal and ethical concerns. See the PLOS Data Policy and FAQ for detailed information.	Yes - all data are fully available without restriction

A Data Availability Statement describing where the data can be found is required at submission. Your answers to this question constitute the Data Availability Statement and **will be published in the article**, if accepted.<br><br>**Important:** Stating 'data available on request from the author' is not sufficient. If your data are only available upon request, select 'No' for the first question and explain your exceptional situation in the text box.<br><br>Do the authors confirm that all data underlying the findings described in their manuscript are fully available without restriction?	
**Describe where the data may be found in full sentences. If you are copying our sample text, replace any instances of XXX with the appropriate details.**<br><br>• If the data are **held or will be held in a public repository**, include URLs, accession numbers or DOIs. If this information will only be available after acceptance, indicate this by ticking the box below. For example: *All XXX files are available from the XXX database (accession number(s) XXX, XXX.).*<br>• If the data are all contained **within the manuscript and/or Supporting Information files**, enter the following: *All relevant data are within the manuscript and its Supporting Information files.*<br>• If neither of these applies but you are able to provide **details of access elsewhere**, with or without limitations, please do so. For example:<br><br>*Data cannot be shared publicly because of [XXX]. Data are available from the XXX Institutional Data Access / Ethics Committee (contact via XXX) for researchers who meet the criteria for access to confidential data.*<br><br>*The data underlying the results presented in the study are available from (include the name of the third party*	Files will be available after acceptance

and contact information or URL).<br>• This text is appropriate if the data are owned by a third party and authors do not have permission to share the data.<br><br>* typeset	
Additional data availability information:	Tick here if the URLs/accession numbers/DOIs will be available only after acceptance of the manuscript for publication so that we can ensure their inclusion before publication.

Manuscript
Click here to access/download;Manuscript;A_Salzberg_Manuscript.docx

The impact of land use change on intraspecific variation in pollinators and herbivores and its effect on strawberry production

Annika Salzberg[1*], Heather Grab[2], Casey Hale[1], Katja Poveda[1]

* Corresponding author: as3565@cornell.edu

1. Cornell University Department of Entomology. 4117 Comstock Hall, Ithaca, NY 14850 USA
2. Cornell University School of Integrative Plant Science. 119 Plant Science Building, Ithaca, NY 14850 USA

<u>Author Contributions</u>

HG and KP conceived and designed the experiments, AS modified experimental design. AS and CH performed the experiments and collected data, AS analyzed the data, AS wrote the manuscript; all authors provided editorial advice.

Abstract

Land use change affects both pollinator and herbivore populations with consequences for crop production. Recent evidence also shows that land use change affects insect traits, with intraspecific body size of pollinators changing across landscape gradients. However, the consequences on crop production of trait changes in different plant interactors have not been well-studied. We hypothesized that changes in body size of key species can be enough to affect crop productivity, and therefore looked at how the field-realistic variation in body size of both an important pollinator, *Bombus impatiens*, and a key pest herbivore, *Lygus lineolaris*, can affect fruit size and damage in strawberry. First, we determined if herbivores, like pollinators, also vary in body size along land use gradients; and second, we tested under controlled conditions how the individual and combined changes in size of an important pollinator and a key herbivore pest affect strawberry fruit production. The key herbivore pest was smaller in landscapes with more natural and semi-natural habitat, confirming that herbivore functional traits can vary along a land use gradient. Additionally, herbivore size, and not pollinator size, marginally affected fruit production - with plants exposed to larger pests producing smaller fruits. Our findings suggest that land use changes at the landscape level affect crop production not just through changes in the taxonomic diversity of insect communities that interact with the plant, but also through changes in body size traits.

Key words: functional traits, landscape composition, pollination, herbivory, ecosystem services, *Fragaria x ananassa*

Introduction

Agriculture is the largest land use type in the world, comprising 40% of the earth's arable land [1]. This percentage continues to rise as natural lands are cleared for use as cropland or pasture [2]. Declines in natural lands threaten biodiversity and the essential ecosystem services wildlife within those habitats provide. Maintaining natural lands in the areas surrounding farmland provides critical services, such as pollination or pest suppression [3,4]. Exploring the effects of landscape simplification on insects that provide crucial ecosystem services is essential to the protection of our global food supply.

Land use change has a variety of local effects, such as increasing insect species richness and abundance in more complex landscapes [3,5–7]. A key effect land use change has on arthropods is its influence on insect traits not just at the community level [8], but also at the population level. Studies have shown a variety of effects of land use change on intraspecific body size. In pollinators such as bees, body size often decreases with increased landscape simplification [9,10]. This effect can be driven by a low availability of resources that results in smaller pollinators, or due to an advantage in being smaller in lower-quality landscapes with fewer resources [9–12]. Pollinators with larger body sizes have been shown to provide better pollination services, with larger bees able to better pollinate plants thereby increasing production of pollination-dependent crops, through increased yield or larger fruits [13].

Fluctuating body size of herbivores, on the other hand, has the potential to also change the effect they have on crops. Smaller herbivores potentially cause less damage to crops than larger herbivores. However, body size of herbivores has not been studied in the context of land use

change, thus it is unknown whether landscape composition affects them, and subsequently whether this affects crop damage. Gaston & Lawton [14] found that body size of small insect herbivores can vary more widely than large herbivore species in the same ecosystem, and that their populations are more variable. Additionally, smaller insect herbivores may be less able to move across landscapes with fewer resources [15]. Studies have found a variety of responses of insect herbivores to changing landscapes [16] in terms of abundance and species richness, though none have focused on body size in particular. Body size trends for pollinators, and the yet unknown body size trends for herbivores, can both have significant implications for the ecosystem services these insects provide. Keeping natural areas preserved around a farm to increase landscape complexity has great potential to affect body size of multiple insect guilds. Exploring the effects of those potential body size changes is crucial, as they have the potential for both positive and negative contributions to ecosystem services and yields.

Here, we examine the interactive effects of a key pest and an important pollinator on fruit size. We expected to find similar results for our pollinator species as prior work - that larger pollinators would provide greater pollination services. For our pest species, we predicted that larger individuals would have greater capacity to cause damage. As both pollinators and herbivores can affect development - and thus the marketability - of many crops, finding ways to mitigate damage caused by both poor pollination and pest feeding is of particular importance. Specifically, we sought to answer four questions: first, does surrounding land use impact intraspecific traits like body size in agriculturally important herbivores? Second, does this size variation result in differential fruit damage - in other words, do larger herbivores cause more

damage? Third, does pollinator body size have an effect on fruit size? And finally, do pollinator and herbivore body size variation have a combined or synergistic effect on fruit size?

Methods

Study system

Strawberries are an important crop in the US, comprising 20% of the world's production [17]. As of 2017, New York State had 1,229 acres of strawberry crop over 568 farms [18]. This accounts for 2% of the total US acreage but the third highest number of individual farms producing strawberries (with California having the most and Pennsylvania the second) [19]. Of these NYS farms, 85% have under 5 acres of strawberry, and 57% have under 1 acre [18]. Most farms growing strawberries in the Northeast US don't grow exclusively strawberries - instead choosing to grow a diverse range of crops along with them.

Strawberries and their interacting pollinators and herbivores are an ideal system to study the effects of landscape simplification on ecosystem services, mediated by changes in the functional traits of herbivores and pollinators. Strawberries are a high-value crop generally grown with high levels of inputs and are heavily managed [20]. Any amount of damage to fruits can render them unmarketable - and fruits can undergo damage in numerous ways. Two of the most important insect-related damages are inadequate pollination by bees (which results in seed underdevelopment) and the characteristic "cat-facing" damage caused when fed on by the tarnished plant bug, *Lygus lineolaris*. Tarnished plant bugs are a generalist herbivorous pest that cause damage to a wide range of crops – in strawberry specifically, they feed on developing

fruits, just as the petals have fallen off the flower, which impedes proper development of those fed-upon achenes (Grab et al. 2018).

In strawberries, fruit weight can provide an accurate estimate of pollination rate and herbivore feeding, as strawberries are an aggregate accessory fruit comprising as many as 300 individual achenes [21]. Each achene must be fertilized and remain undamaged in order for the surrounding tissue to develop without major malformations that reduce overall yield and marketability [22]. Hence, the weight of a fruit is highly correlated with the number of pollinated, undamaged achenes [23]. An average of four pollinator visits per flower is required to achieve full pollination and maximum fruit weight [24], but densities as low as a single *Lygus* nymph per flower cluster can reduce fruit weight by 20% [22].

Tarnished plant bug size across the landscape gradient

In summer 2019, 11 sites in the Finger Lakes region of New York State were selected across a landscape gradient. The landscape surrounding these sites consisted of 15% to 42% natural area at a 750m radius. Each site was visited once between August 12-30, and three of those sites were also visited once on July 2-3, though as tarnished plant bug populations were not large enough to enable enough specimens to be caught at that time, most individuals were caught in mid-late August. Landscape composition within a 500, 750, and 1000 meter radius around each farm was determined via the 2019 USDA NASS CropScape Cropland Data Layer (nassgeodata.gmu.edu/CropScape). Categories of land cover quantified were agricultural, agricultural with pasture, natural forested, natural open, pasture, and urban cover, consolidated from the smaller categories available from the Data Layer. At each site, tarnished plant bugs were collected, and their body size measured, using pronotum width at its widest point as a proxy for whole body size [25]. Size was measured using an Olympus SZX10 stereo microscope and

DP22 microscope digital camera, with Olympus CELLSENS Standard v.1.16 software used to measure bugs to the nearest 10 µm.

Effect of bee size and tarnished plant bug size on strawberry fruit size

A greenhouse bioassay was conducted in spring 2021 to examine the effects of bee and tarnished plant bug size on strawberry pollination and damage, respectively. A total of 300 strawberry plants (*Fragaria x ananassa*, variety: Seascape, bare root plants sourced from Nourse Farms) were grown in the greenhouse (14 hours daylight, 70-74°F day/70-66°F night) until they flowered.

Three managed *Bombus impatiens* bumble bee colonies were sourced from Biobest (Biobest Standard hives). From these three hives, we constructed ten microcolonies (Fig 1) of 8 female workers each, all from the same source colony. Five microcolonies contained individuals with the smallest body sizes, selected by eye, and five contained individuals with the largest body sizes. Microcolonies were allowed to acclimate for one week with supplemental pollen and 30% sugar solution. Post-experiment, bee body size was measured to confirm that large and small colonies were significantly different in size by measuring intertegular distance (ITD), a standard measure of body size in bees [26], Welch two-sample t-test comparing mean sizes of large versus small colonies, t = 6.4746, df = 4.7168, p = 0.001628). To ensure these colonies represented a standard range of bee sizes for the region, we compared them with other commercial hives used in other regional studies. In total, our average bee ITD was 3.47 ± 0.36mm (mean ± 1SD). Miller et al. [27] used commercial hives in the same region of New York State and found an average

bee ITD of 3.34 ± 0.45mm (mean ± 1SD) and in nearby Southern Quebec, Gervais et al. [28] found an average ITD of 3.943 ± 0.318mm (mean ± 1SD).

Fig 1: Diagram of *B. impatiens* microcolony setup. Consists of (1) Entrance/exit tube with ramp, with yellow tape placed around entry and on ramp to guide bees in; (2) Main chamber, plastic with lid on top and perforations for airflow (not pictured), with 8 individual bees; (3) Pollen balls (made in-lab with Arizona sourced pollen granules (CC Pollen High Desert brand) and 30% sucrose solution); (4) Tube with 30% sucrose solution, with small hole at base.

All microcolonies were 'trained' on strawberry plants for a minimum of three days in order to ensure each individual had experience visiting strawberry flowers. Training consisted of colonies being kept open in a 24x36" mesh cage, with three or four blooming strawberry plants. All colonies were kept in a growth chamber set at 14 hours daylight and 70-74°F day temperature, 66-70°F night temperature.

Pollination and herbivory trials were conducted outdoors and in the greenhouse, respectively (Fig 2). Pollination cages (24x36" white mesh pop-ups) were set up outdoors in the morning starting at around 9am each day. Only plants with at least two open secondary flowers were used. Experimental plants were brought from the greenhouse and one or two were carefully inserted into each cage. These plants were then observed until the first visit by a bee to one of the exposed secondary flowers. The plant was then removed, the pollinated flower was marked with yarn, and the other unpollinated secondary flower (at approximately the same stage of development) was marked as unpollinated. Pollination trials were run until around 12 pm each day, after which time visitation activity was negligible. Pollination trials were run for four days,

until at least 60 pollinated plants per pollinator size group were acquired. Once plants were pollinated, they were transported back to the greenhouse and allocated into their *Lygus* trial group.

Fig 2: Flowchart of bumble bee pollination and tarnished plant bug damage trials.

To determine the effect of *Lygus* size on fruit damage and size, we conducted an experiment exposing developing fruits to varying sized *Lygus*. Approximately half of plants from each pollination size group, 30 plants pollinated by large bees and 34 plants pollinated by small bees, were placed in empty cages (no damage treatment). Each of the remaining pollinated plants, 30 pollinated by large bees and 30 pollinated by small bees, were placed in individual 12x12 cages along with a single wild-caught tarnished plant bug individual. These individuals varied in size from 1.930 to 2.328mm (pronotal width) and were randomly selected. This range is similar to the range seen across local landscapes - in 2019, field collections of *Lygus* made across 10 sites of varying landscape complexity showed a range in size from 1.67 to 2.27mm (pronotal width). Due to *Lygus* mortality during trials, the final count of usable plants was 22 plants pollinated by large bees and 29 plants pollinated by small bees. The *Lygus* were left to feed on the plants for 24 hours, at which point they were removed, frozen, and their pronotal width measured to the nearest 10 µm using an Olympus SZX10 stereo microscope and DP22 microscope digital camera, with Olympus CELLSENS Standard v.1.16 software. Plants were then removed from small cages and placed in large ones, which were also kept in large cages to eliminate the risk of stray pollinators in the greenhouse accessing them. Plants were watered regularly and monitored

until fruits were fully developed. Once that stage was reached, each fruit was removed and weighed.

Statistical analysis

Tarnished plant bug size across the landscape gradient

We constructed linear mixed effects models to look at the effects of land cover on *Lygus* size, with pronotal width as the response variable and land cover at 750 meters and sex of the insect as predictors, with farm as a random effect. Models were constructed for all land cover types over all three landscape scales (500, 750, and 1000m), with sex as an additive factor and farm as a random factor. We only report effects on significant land cover variables. In order to determine potential correlations between the two significant land cover types, a Pearson's correlation test was run to determine correlation between urban and forest land cover.

Effect of bee presence/absence and tarnished plant bug presence/absence on strawberry fruit size

To determine whether the presence or absence of pollinators and plant bugs affected final strawberry fruit weight, we constructed a linear mixed effects model with fruit weight as the response variable, and *Lygus* presence/absence and pollination treatment (visited or unvisited) as the two predictors. Microcolony number (which microcolony the bee came from) was used as a random factor.

Effect of bee size and tarnished plant bug size on strawberry fruit size

To determine how tarnished plant bug size and pollinator size affect strawberry fruit weight we constructed a linear mixed effects model with fruit weight as the response variable, and *Lygus*

width and pollinator size treatment as the two predictors. Microcolony number (which microcolony the bee came from) was used as a random factor. This analysis was run on a subset of the data that only included fruits that were both exposed to *Lygus* and had been pollinated. Fruit weight was used as a metric for the amount of damage done by the *Lygus*.

Analyses were conducted in RStudio 2021.09.0 [29], using packages ‘nlme’ [30], and ‘stats’ [31].

Graphs were constructed in RStudio 2021.09.0 [29], using packages ‘ggplot2’ [32] and ‘cowplot’ [33].

Results

Landscape affects tarnished plant bug size

For tarnished plant bugs collected across a landscape gradient, we found that both males and females were larger in landscapes with higher urban and developed land cover ($F_{1,8}$ = 6.552, P = 0.0387, Fig 3a, Supp. Table 1). We also found that tarnished plant bugs from landscapes with higher forested land cover were marginally smaller ($F_{1,8}$ = 3.718, P = 0.0628, Fig 3b, Supp. Table 2). These two land cover types were negatively correlated with each other (Pearson’s correlation, t = -12.416, df = 65, P < 0.001, correlation coefficient=-0.84), thus it is impossible to tease apart if the effect is caused by one or the other. Female *Lygus* were larger than males (Fig 3a: $F_{1,56}$ = 5.294, P = 0.0251; Fig 3b: $F_{1,56}$ = 6.760, P = 0.0119), but their relationship to land cover is the same (urban landcover interaction with sex: $F_{1,55}$ = 0.132, P = 0.7175; forest cover interaction with sex: $F_{1,55}$ = 0.524, P = 0.4722.

Fig 3: Tarnished plant bug pronotal width at widest point across a landscape gradient. Red points/line are female bugs, blue are males. **(A)** Urban land cover, trendlines = linear regressions, shaded area = 95% confidence area. **(B)** Forested land cover, trendlines = linear regressions, shaded area = 95% confidence area.

Bee presence, but not tarnished plant bug presence, affected strawberry fruit size

Fruit weight differed significantly between pollinated and unpollinated flowers ($F_{1,198} = 38.046$, $P < 0.001$, Fig. 4a). For all plants (both pollinated and unpollinated) *Lygus* presence did not affect fruit weight ($F_{1,198} = 0.0255$, $p = 0.873$, Fig. 4b, Supp. table 3).

Fig 4: (A) Fruit weight in grams for flowers that were visited or not visited by a bumble bee (***=p<0.001) **(B)** For all plants both pollinated and unpollinated, fruit weight in grams by presence or absence of tarnished plant bugs. Each plant was exposed to one individual for 24 hours. (p=0.817).

Tarnished plant bug size, and not pollinator size, affected strawberry fruit size

We found that there was no difference in the weight of fruits pollinated by large and small bees ($F_{1,40} = 0.2424$, $P = 0.6252$, Fig 5a). Because we found that *Lygus* size varies across the landscape gradient, we wanted to know if *Lygus* size affected the amount of damage they inflict on strawberries. We found that among pollinated fruits, exposure to larger tarnished plant bugs caused fruits to be marginally smaller ($F_{1,40} = 3.57$, $P = 0.0661$, Fig 5b). Interestingly, there was no interaction between herbivore size and bee size ($F_{1,39} = 0.7082$, $P = 0.4052$, Supp. Fig A), indicating that effect of tarnished plant bug size on resulting fruits did not vary depending on the size of the pollinator.

Fig 5: (A) Fruit weight by size of bumble bee visitor (p=0.6252); **(B)** Fruit weight by size of tarnished plant bug the plant was exposed to for 24 hours (p=0.0661). Both A and B only include data from fruits that were pollinated.

Discussion

This study sought to untangle the effects of land-use change on ecosystem services and disservices, as mediated by changes in intraspecific body size of an important pollinator and a key pest. We then asked whether those changes in ecosystem services and disservices provided by the two focal species would combine or synergize to create a greater positive or negative impact on fruit size.

First, we wanted to elucidate the effects of landscape composition on herbivore size. We found that tarnished plant bugs were smaller in landscapes with more natural forest and less urban area in the surrounding 750m around strawberry farms. This is an important finding, as there are virtually no studies looking at how intraspecific herbivore body size varies across a landscape gradient. Our results for this herbivore pest also contrast with studies done on pollinators, many of which have found increasing body sizes when landscape complexity increases [10,34]. Tarnished plant bugs could be using urban areas, but since the gradient is so small (0.05-8.81 %) this result is more likely a response to the much broader forest gradient (6.52-39.19 %). There are many possible reasons for finding smaller *Lygus* in more complex landscapes; some of these include potentially increased competition for resources [15], more natural enemies putting pressure on the pest population (though patterns vary [35,36]), or fewer crop resources available for feeding. Additionally, tarnished plant bugs utilize perennial woody and forest edge habitat

not only as an alternative area for suitable hosts but also for overwintering [37]. Larger individuals with greater dispersal capacity [38] may move more widely across the landscape than their smaller counterparts which may be constrained to areas near alternative hosts and overwintering sites.

Having confirmed that landscape composition affects *Lygus* size, we then wanted to know whether the size of the bug would differentially affect the growth of the developing fruit it was feeding on, resulting in smaller fruits. While prior studies have suggested a correlation between bee size and pollination efficiency [13,39], though we are unaware of any studies evaluating the same patterns in herbivores. We saw a trend that the presence of larger bugs resulted in smaller fruit regardless of pollination treatment. Fruit size went from ~13 grams when exposed to small bugs to ~7.5 grams when exposed to large bugs, a decrease of over 40%, though this effect was only marginally significant. Even as a marginal effect, a 40% decrease in weight translates to important yield losses when scaled across an entire crop field. The effects we report are also likely to be conservative estimates, as our fruits were exposed to just a single bug over only 24 hours, while in the field, the fruit would be exposed for longer periods of time and potentially to more bugs.

There is notably no difference in fruit size between fruits exposed or not exposed to *Lygus*. Though we find their presence/absence to result in the same range of fruit sizes, when the bugs *are* present, their size variation contributed to explaining the variation in fruit sizes observed with marginally smaller pollinated fruits produced in the presence of larger *Lygus*. Although it is unclear why we don't see a negative effect of overall *Lygus* presence, we see that there is a huge variation in fruit weight in our experiment, which could be caused by differences in pollination

quality (due to limited pollen load on the bees from single visits) or fruit development. However, part of that variation seems to be explained by the variation in *Lygus* size. In field-realistic settings we may see a much wider range of fruit size changes - and it is unclear whether pollination and *Lygus* damage will scale up at the same rate suggesting an important direction for future research.

As many previous studies in different crops system have shown, bee size (both inter- and intra-specific) is frequently an important factor impacting fruit set and size [13,40,41]. Contrary to these prior studies, we found no difference in fruit weight between flowers pollinated by large versus small bees. This suggests that for this cultivar of strawberry and its interaction with this particular species of pollinator, *Bombus impatiens,* intraspecific body size of the pollinator does not matter. This result could also be due to the single visit nature of this study, where pollen loads on the bees were likely to be lower than in other studies where bees were allowed unlimited visitation. Pollen load could interact with other traits like hairiness, position of pollen loads, behaviors, and flower traits to influence pollination outcomes [42–45].

Our results support the large body of literature showing the benefits of natural areas for agricultural production. We show that the presence of natural areas around a farm can lead to a decrease in the body size of herbivores with consequences for crop yield. Alternate methods of pest suppression, such as the maintenance of land cover diversity, have the potential to lessen the usage of pesticides and lower costs for growers. Previous work in the region has shown how greater landscape complexity also has a wide variety of positive impacts on pollinators - including increased bee richness, abundance, larger bee body size, and increased pollination

[34,46] - as well as decreases in *Lygus* abundance [47]. The benefits to pollinators and the potential for decreased herbivory could provide double benefits to growers whose farms are situated in more complex landscapes. Strawberry growers should consider keeping some forested land around their fields, as prioritizing the maintenance of a diverse/complex landscape has the potential to provide benefits for both pollination services and pest suppression alike.

Funding

This work was supported by USDA-NIFA grant #2019-67013-29367.

Conflict of Interest

The authors declare that they have no conflict of interest.

Acknowledgments

Huge thanks to all the people who helped with bumble bee single visit trials - Hayley Schroeder, Dr. Jules Davis, and Dr. Laura Figueroa. Thanks as well to the Cornell Greenhouse staff for plant care and support. Thanks to our funders at USDA-NIFA for supporting our research.

References

1. Seto KC, Fragkias M, Güneralp B, Reilly MK. A Meta-Analysis of Global Urban Land Expansion. Añel JA, editor. PLoS ONE. 2011 Aug 18;6(8):e23777.

2. Green R, Cornell S, Scharlemann J, Balmford A. Farming and the Fate of Wild Nature. Science. 2005 Feb 1;307:550–5.

3. Batáry P, Báldi A, Kleijn D, Tscharntke T. Landscape-moderated biodiversity effects of agri-environmental management: a meta-analysis. Proc Biol Sci. 2011 Jun 22;278(1713):1894–902.

4. Kremen C, Miles A. Ecosystem Services in Biologically Diversified versus Conventional Farming Systems: Benefits, Externalities, and Trade-Offs. Ecol Soc [Internet]. 2012 [cited 2023 Nov 30];17(4). Available from: https://www.jstor.org/stable/26269237

5. Chaplin-Kramer R, O’Rourke ME, Blitzer EJ, Kremen C. A meta-analysis of crop pest and natural enemy response to landscape complexity. Ecol Lett. 2011 Sep;14(9):922–32.

6. Tscharntke T, Tylianakis JM, Rand TA, Didham RK, Fahrig L, Batáry P, et al. Landscape moderation of biodiversity patterns and processes - eight hypotheses. Biol Rev. 2012 Aug;87(3):661–85.

7. Dainese M, Martin EA, Aizen MA, Albrecht M, Bartomeus I, Bommarco R, et al. A global synthesis reveals biodiversity-mediated benefits for crop production. Sci Adv. 2019 Oct 16;5(10):eaax0121.

8. Gámez-Virués S, Perović DJ, Gossner MM, Börschig C, Blüthgen N, de Jong H, et al. Landscape simplification filters species traits and drives biotic homogenization. Nat Commun. 2015 Oct 20;6(1):8568.

9. Chole H, Woodard SH, Bloch G. Body size variation in bees: regulation, mechanisms, and relationship to social organization. Curr Opin Insect Sci. 2019 Oct;35:77–87.

10. Renauld M, Hutchinson A, Loeb G, Poveda K, Connelly H. Landscape Simplification Constrains Adult Size in a Native Ground-Nesting Bee. PLOS ONE. 2016 Mar 4;11(3):e0150946.

11. Oliveira MO, Freitas BM, Scheper J, Kleijn D. Size and Sex-Dependent Shrinkage of Dutch Bees during One-and-a-Half Centuries of Land-Use Change. PLOS ONE. 2016 Feb 10;11(2):e0148983.

12. Gérard M, Marshall L, Martinet B, Michez D. Impact of landscape fragmentation and climate change on body size variation of bumblebees during the last century. Ecography. 2021;44(2):255–64.

13. Jauker F, Speckmann M, Wolters V. Intra-specific body size determines pollination effectiveness. Basic Appl Ecol. 2016 Dec 1;17:714–9.

14. Gaston KJ, Lawton JH. Patterns in Body Size, Population Dynamics, and Regional Distribution of Bracken Herbivores. Am Nat. 1988 Nov;132(5):662–80.

15. Tscharntke T, Brandl R. Plant-Insect Interactions in Fragmented Landscapes. Annu Rev Entomol. 2004;49(1):405–30.

16. Rossetti MR, Tscharntke T, Aguilar R, Batáry P. Responses of insect herbivores and herbivory to habitat fragmentation: a hierarchical meta-analysis. Ecol Lett. 2017;20(2):264–72.

17. Samtani J, Rom C, Friedrich H, Fennimore S, Finn C, Petran A, et al. The Status and Future of the Strawberry Industry in the United States. HortTechnology. 2019 Jan 31;29:1–14.

18. USDA - National Agricultural Statistics Service - 2017 Census of Agriculture - Volume 1, Chapter 1: State Level Data, Table 33 [Internet]. [cited 2023 Nov 30]. Available from: https://www.nass.usda.gov/Publications/AgCensus/2017/Full_Report/Volume_1,_Chapter_1_State_Level/New_York/

19. USDA - National Agricultural Statistics Service - 2017 Census of Agriculture - Volume 1, Chapter 1: State Level Data, Table 38 [Internet]. [cited 2023 Nov 30]. Available from:

https://www.nass.usda.gov/Publications/AgCensus/2017/Full_Report/Volume_1,_Chapter_1_State_Level/New_York/

20. Pritts M, Handley D, Walker C, Boyce B, Cooley D, Daum D, et al. Strawberry Production Guide for the Northeast, Midwest, and Eastern Canada (NRAES-88). 1998 [cited 2023 Nov 30]; Available from: https://hdl.handle.net/1813/66932

21. Webb RA, Terblanche JH, Purves JV, Beech MG. Size factors in strawberry fruit. Sci Hortic. 1978 Dec 1;9(4):347–56.

22. Schaefers GA. Yield Effects of Tarnished Plant Bug Feeding on June-Bearing Strawberry Varieties in New York State12. J Econ Entomol. 1980 Oct 1;73(5):721–5.

23. Webb RA, Purves JV, White BA. The components of fruit size in strawberry. Sci Hortic. 1974 Jun 1;2(2):165–74.

24. Chagnon M, Gingras J, De Oliveira D. Effect of Honey Bee (Hymenoptera: Apidae) Visits on the Pollination Rate of Strawberries. J Econ Entomol. 1989 Oct 1;82(5):1350–3.

25. Brent CS. Stage-specific effects of population density on the development and fertility of the western tarnished plant bug, Lygus hesperus. J Insect Sci. 2010 Jan 1;10(1):49.

26. Kendall LK, Rader R, Gagic V, Cariveau DP, Albrecht M, Baldock KCR, et al. Pollinator size and its consequences: Robust estimates of body size in pollinating insects. Ecol Evol. 2019;9(4):1702–14.

27. Miller O, Hale C, Richardson L, Sossa D, Iverson A, McArt S, et al. Commercial Bombus impatiens colonies function as ecological traps for wild queens. J Appl Ecol. 2023;60(4):592–600.

28. Gervais A, Courtois È, Fournier V, Bélisle M. Landscape composition and local floral resources influence foraging behavior but not the size of Bombus impatiens Cresson (Hymenoptera: Apidae) workers. PLOS ONE. 2020 Jun 25;15(6):e0234498.

29. R Core Team. R: A language and environment for statistical computing [Internet]. Vienna, Austria: R Foundation for Statistical Computing; 2020. Available from: https://www.R-project.org/.

30. Pinheiro J, Bates D, R Core Team. nlme: Linear and Nonlinear Mixed Effects Models. R package version 3.1-157 [Internet]. Vienna, Austria: R Foundation for Statistical Computing; 2022. Available from: <https://CRAN.R-project.org/package=nlme>

31. R Core Team. Package: “stats” [Internet]. Vienna, Austria: R Foundation for Statistical Computing; 2020. Available from: https://www.R-project.org/.

32. Wickham J. ggplot2: Elegant Graphics for Data Analysis. New York, 2016. [Internet]. New York: Springer-Verlag; 2016. Available from: https://cran.r-project.org/web/packages/ggplot2/

33. Wilke CO. cowplot: Streamlined Plot Theme and Plot Annotations for “ggplot2”. R package version 1.1.1. [Internet]. New York: Springer-Verlag; 2020. Available from: https://CRAN.R-project.org/package=cowplot

34. Grab H, Brokaw J, Anderson E, Gedlinske L, Gibbs J, Wilson (Tuell) J, et al. Habitat enhancements rescue bee body size from the negative effects of landscape simplification. J Appl Ecol. 2019 Jun 1;56.

35. Menalled FD, Marino PC, Gage SH, Landis DA. Does Agricultural Landscape Structure Affect Parasitism and Parasitoid Diversity? Ecol Appl. 1999;9(2):634–41.

36. Landis DA, Wratten SD, Gurr GM. Habitat Management to Conserve Natural Enemies of Arthropod Pests in Agriculture. Annu Rev Entomol. 2000;45(1):175–201.

37. Sheffield CS. Overwintering biology of the tarnished plant bug, Lygus lineolaris (Palisot de Beauvois)(Hemiptera: Miridae), in Nova Scotia, and the potential use of ice nucleating active bacteria for insect pest control [Internet]. [Ottawa]: Acadia University; 1999 [cited 2023 Nov 30]. Available from: https://library-archives.canada.ca/eng/services/services-libraries/theses/Pages/item.aspx?idNumber=46580717

38. Shrestha G, Wiman NG, Rondon SI. Flight Potential of Western Tarnished Plant Bug (Hemiptera: Miridae). Furlong M, editor. J Econ Entomol. 2022 Feb 9;115(1):93–100.

39. Ricketts TH, Regetz J, Steffan-Dewenter I, Cunningham SA, Kremen C, Bogdanski A, et al. Landscape effects on crop pollination services: are there general patterns? Ecol Lett. 2008;11(5):499–515.

40. Hoehn P, Tscharntke T, Tylianakis JM, Steffan-Dewenter I. Functional group diversity of bee pollinators increases crop yield. Proc R Soc B Biol Sci. 2008 Jul;275(1648):2283–91.

41. Martins KT, Gonzalez A, Lechowicz MJ. Pollination services are mediated by bee functional diversity and landscape context. Agric Ecosyst Environ. 2015 Feb 1;200:12–20.

42. Müller A. Morphological Specializations in Central European Bees for the Uptake of Pollen from Flowers with Anthers Hidden in Narrow Corolla Tubes (Hymenoptera: Apoidea). Entomol Gen. 1995 Sep 1;20(1–2):43–57.

43. Rader R, Edwards W, Westcott DA, Cunningham SA, Howlett BG. Pollen transport differs among bees and flies in a human-modified landscape. Divers Distrib. 2011;17(3):519–29.

44. Nicholls E, Hempel de Ibarra N. Assessment of pollen rewards by foraging bees. Funct Ecol. 2017;31(1):76–87.

45. Brunet J, Thairu MW, Henss JM, Link RI, Kluever JA. The Effects of Flower, Floral Display, and Reward Sizes on Bumblebee Foraging Behavior When Pollen Is the Reward and Plants Are Dichogamous. Int J Plant Sci. 2015 Nov;176(9):811–9.

46. Grab H, Branstetter MG, Amon N, Urban-Mead KR, Park MG, Gibbs J, et al. Agriculturally dominated landscapes reduce bee phylogenetic diversity and pollination services. Science. 2019 Jan 18;363(6424):282–4.

47. Grab H, Danforth B, Poveda K, Loeb G. Landscape simplification reduces classical biological control and crop yield. Ecol Appl. 2018;28(2):348–55.

Figure1

Click here to access/download;Figure;Fig1.tiff

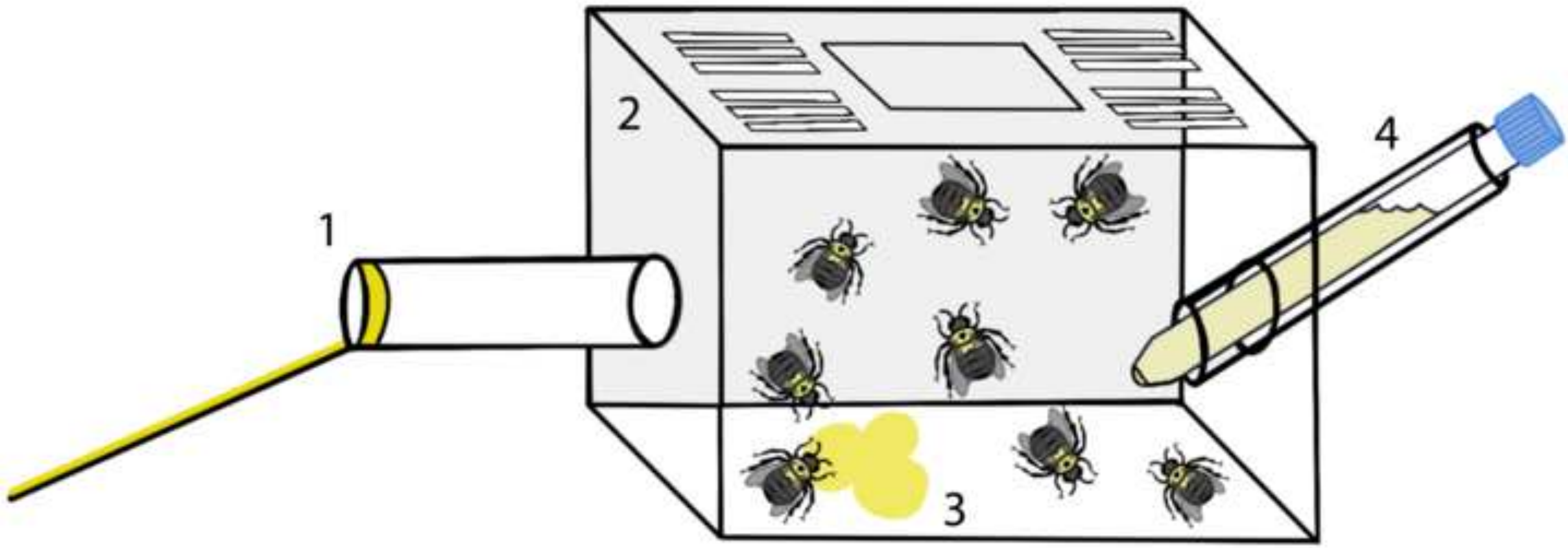

Figure2

Click here to access/download;Figure;Fig2.tiff

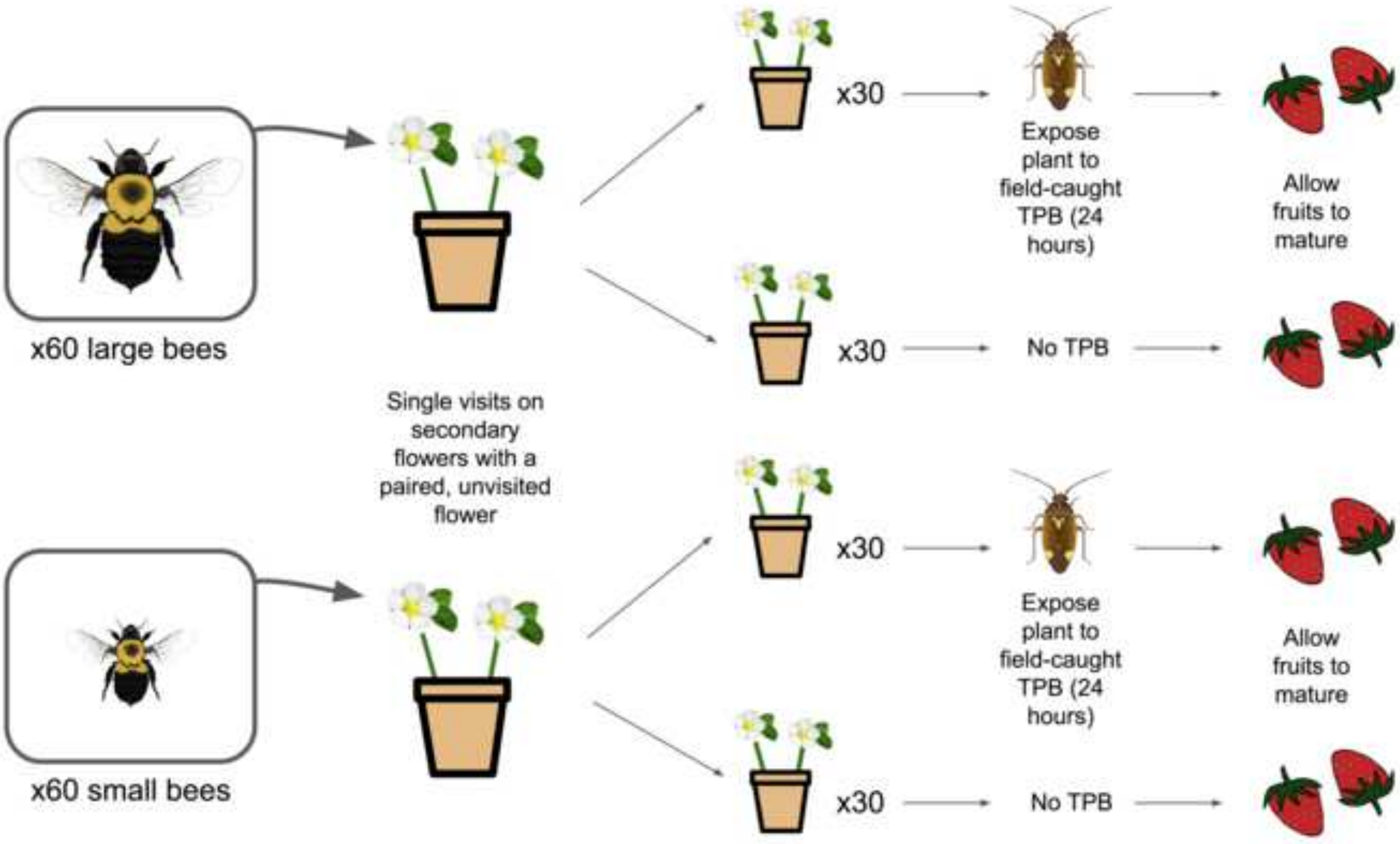

Figure3

Click here to access/download;Figure;Fig3.tiff

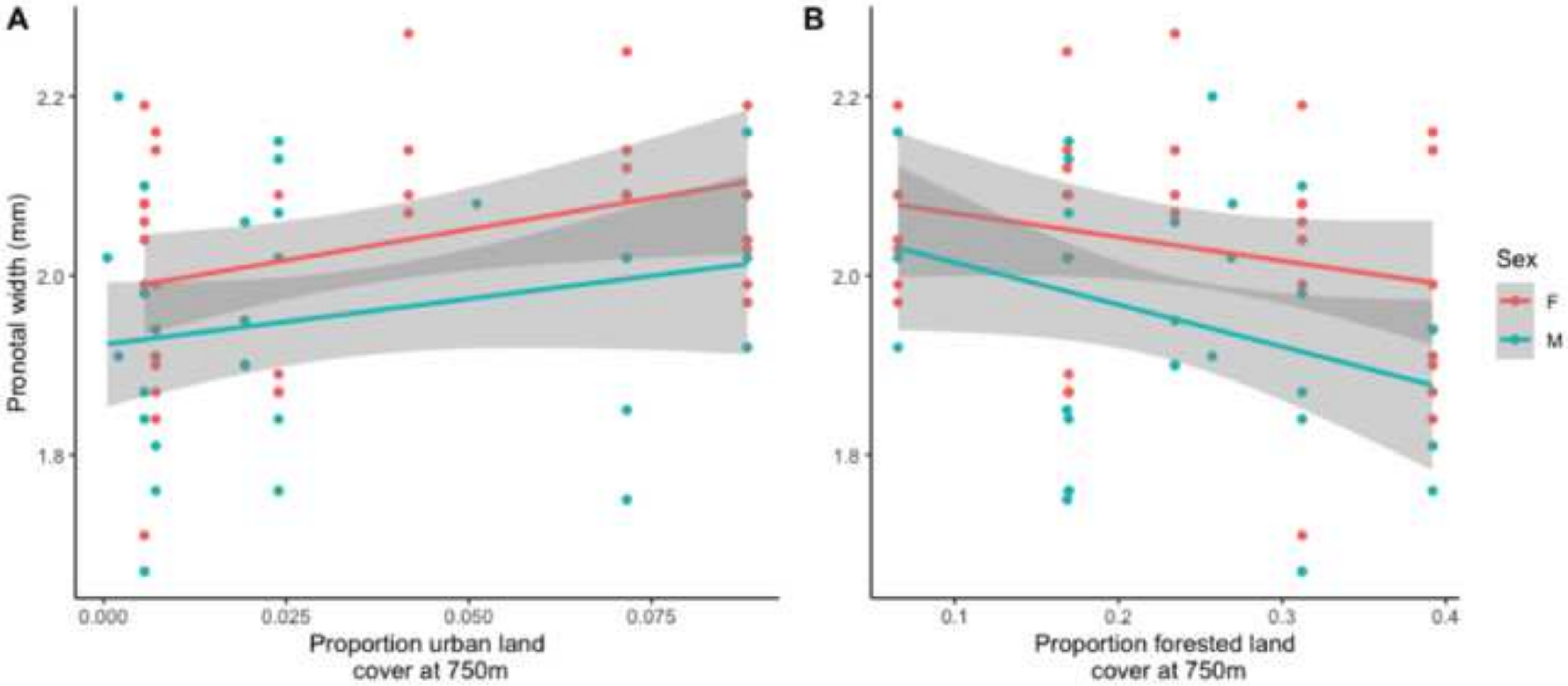

Figure4

Click here to access/download;Figure;Fig4.tiff

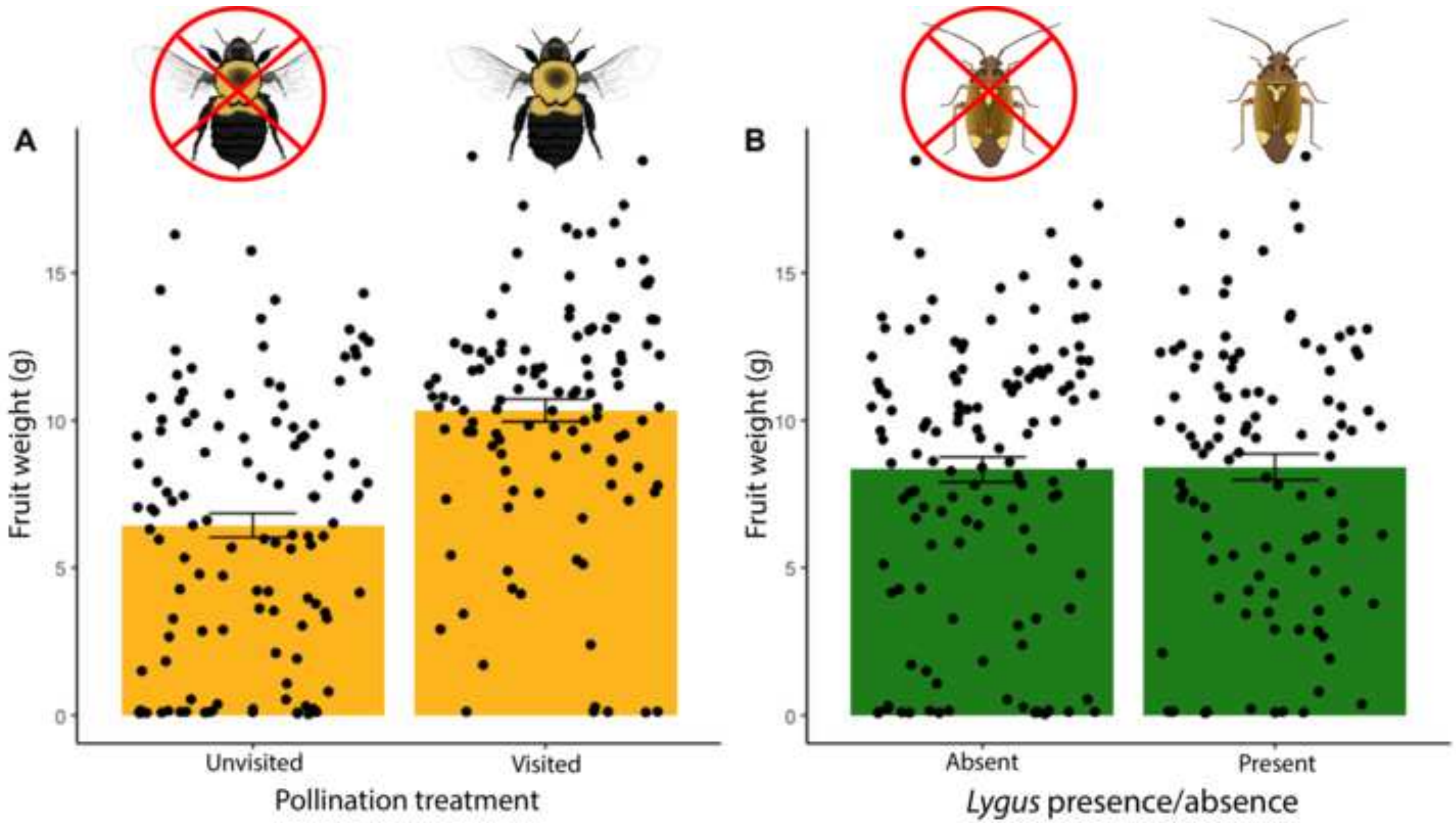

Figure5

Click here to access/download;Figure;Fig5.tiff

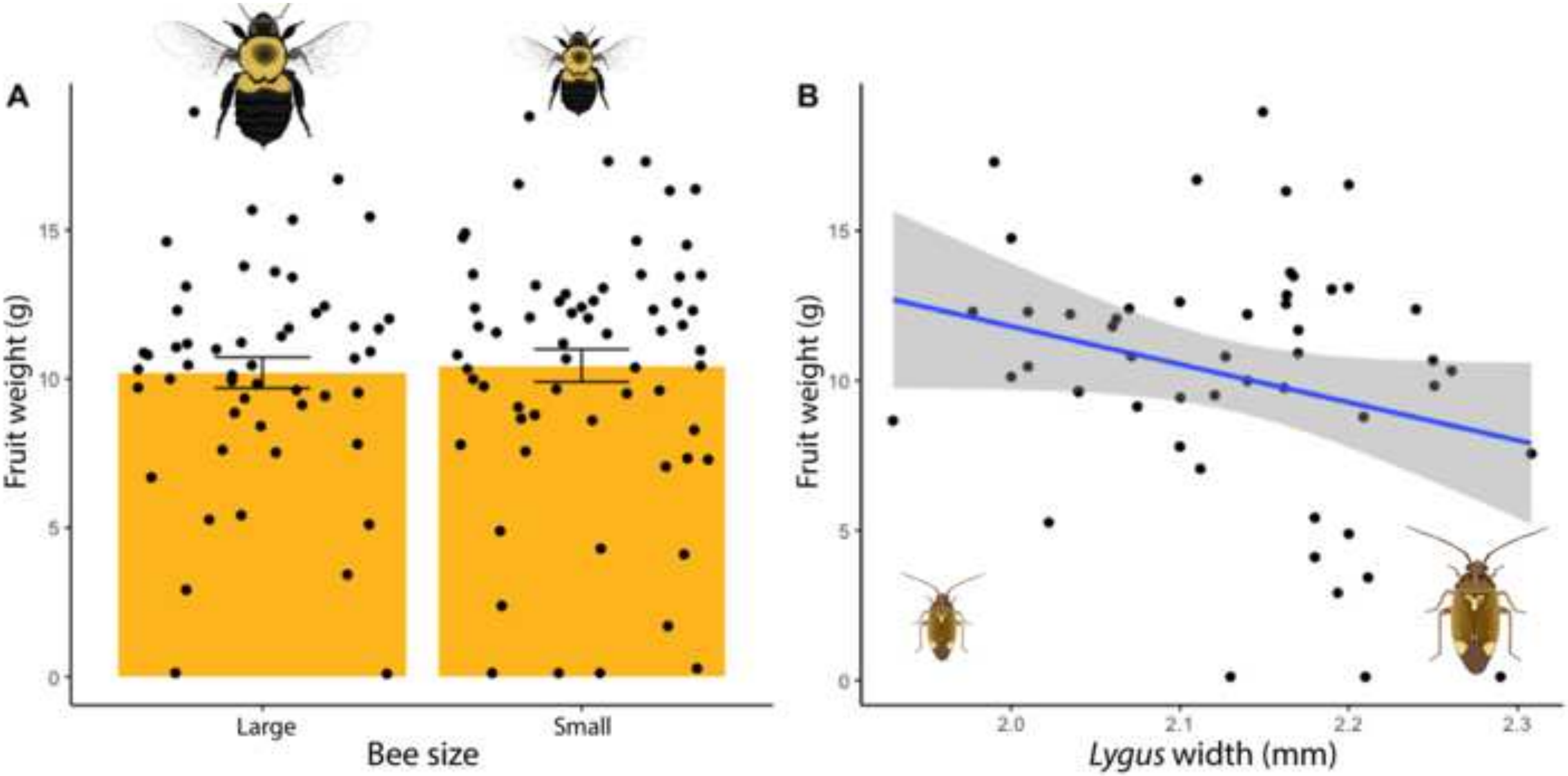

Supplement

Click here to access/download
Supporting Information
A_Salzberg_Supplement.docx
