# Supplementary material for: Herbivore and pollinator body size effects on strawberry fruit quality
Source: PLoS One. 2024 Jun 25;19(6):e0305370. doi: 10.1371/journal.pone.0305370 (PMC11198852; doi:10.1371/journal.pone.0305370)
Supplement: Attachment — Submitted filename: Response to Reviewers.docx [file pone.0305370.s006.docx]

Dear Dr. Ghasemi-Soloklui and PLOS ONE reviewers,

We would like to thank you all for your careful review and valuable comments on our manuscript! We feel that your guidance has helped significantly improve our work. Below, please find our responses to all points raised by reviewers.

Reviewer #1: I have read this new version of the manuscript and the responses to the comments. I thank the authors for their great job addressing concerns. The manuscript is substantially improved and I believe it will make a great contribution to Plos One.
The only concern I have is the new version of the title. I think that the title "Larger pests but not pollinators impact strawberry fruit quality" is a bit misleading and gives the impression that animal pollination doesn’t matter for strawberry, which is contrary to the results of this study and the literature.

*Thank you for voicing your concern on the title – we agree, the grammar of the title was unclear, and we have modified the title to remove the ambiguity that might imply that animal pollination isn’t important for strawberry. We hope the new title, “*Herbivore and pollinator body size effects on strawberry fruit quality” *is a better representation of the paper and that this is acceptable to the reviewer.*

Please note that funding information should not appear in any section or other areas of your manuscript. We will only publish funding information present in the Funding Statement section of the online submission form. Please remove any funding-related text from the manuscript.

*We have removed the funding information section from the manuscript, thank you for pointing this out!*

Thank you!

Best regards,

Annika Salzberg
